# Aggravation of acute kidney injury by mPGES-2 down regulation is associated with autophagy inhibition and enhanced apoptosis

**DOI:** 10.1038/s41598-017-10271-8

**Published:** 2017-08-31

**Authors:** Ting Li, Ying Liu, Jie Zhao, Shuying Miao, Yunfei Xu, Ke Liu, Meidong Liu, Guiliang Wang, Xianzhong Xiao

**Affiliations:** 1 Department of Pathophysiology, Xiangya School of Medicine, Central South University, Changsha, 410078 China; 2 Department of Physiology, Changzhi Medical College, Changzhi, 046000 China; 3 Department of Neurosurgery, Xiangya Hospital, Central South University, Changsha, 410078 China; 4 Department of Digestive Internal Medicine, Gannan Medical University Pingxiang Hospital, Pingxiang, 337055 China

## Abstract

The deletion of microsomal prostaglandin E synthase-2 (mPGES-2) does not affect *in vivo* PGE_2_ production, and the function of this enzyme remains unknown until now. This study investigated the expression and roles of mPGES-2 in LPS induced acute kidney injury (AKI) both *in vitro* and *in vivo*. We found that mPGES-2 was up-regulated in kidney of mice with LPS induced AKI. Inhibition of mouse mpges2 gene expression exacerbated LPS-induced renal dysfunction, renal tubular cell damage and apoptosis, while inhibited kidney autophagy. Further cellular experiments showed that over-expression of mPGES-2 resulted in increased autophagy and decreased apoptosis rate of renal tubular epithelial cells. In addition, treatment with autophagy inhibitor 3-methyladenine could reverse the above-mentioned results. On the contrary, interference of mPGES-2 expression by siRNA decreased autophagy level but significantly increased apoptosis of tubular epithelial cells and treatment with autophagy inducer rapamycin can reverse these results. Overall, our study shows that mPGES-2 can protect renal tubular epithelial cells by regulating autophagy levels and aggravation of acute kidney injury by mPGES-2 down regulation is associated with autophagy inhibition and enhanced apoptosis.

## Introduction

Acute kidney injury (AKI) has a high incidence in critically ill patients and is a serious complication of sepsis^1, 2^. In recent years, with the early diagnosis and the level of renal replacement therapy continuously improving, prognosis of AKI patients has improved. But it is noteworthy that compared with that of patients with non-sepsis-induced AKI and patients with simple sepsis, the mortality of patients with sepsis-induced AKI is significantly higher, maintaining at 50% to 70%^3, 4^. Such a high mortality in patients with sepsis-induced AKI is closely related with lack of understanding of its underlying mechanisms. Therefore, further elucidating the pathogenesis of sepsis-induced AKI is very important.

Autophagy is a lysosome-mediated catabolism process. Although autophagy is rare in the physiological situation, it can be significantly activated to maintain cell homeostasis and ensure cell energy supply under stress or pathological conditions such as starvation, hypoxia, infection and tumor^5–7^. Numerous studies have shown that autophagy is activated in renal tubular epithelial cells upon occurrence of AKI, and activation of autophagy has a protective effect on AKI model animals^8–11^. However, the key signaling pathways involved in the induction and regulation of autophagy in AKI have not been elucidated. Therefore, in-depth studies on the mechanisms of autophagy induction and action will contribute to the prevention and treatment of sepsis-induced AKI.

Microsomal prostaglandin E synthase Type 2 (mPGES-2) is a new type of prostaglandin synthase purified from bovine heart by Watanabe *et al*.^12^ and was cloned in 2002 by Tonkawa *et al*.^13^. Early studies have shown that mPGES-2 is mainly expressed in heart, brain, kidney and small intestine^14^. *In vitro* studies have shown that mPGES-2 has prostaglandin E_2_ (PGE_2_) synthesis activity^15^. However, *in vivo* experiments showed that knockout of mPGES-2 did not affect the synthesis of mouse PGE_2_^16^. In addition, it was reported that mPGES-2 can only catalyze PGE_2_ synthesis in the free state as an enzyme, while *in vivo* it forms a complex with heme and does not participate in PGE_2_ synthesis^17, 18^. A recent study has shown that knockout of mPGES-2 does not affect PGE_2_ the synthesis, but aggravates STZ-induced liver injury in mice^19^, suggesting that mPGES-2 may play an important role independent of PGE_2_. Therefore, the role of mPGES-2 *in vivo*, especially its physiological and pathological features need to be further explored.

Normally, mPGES-2 is mainly located in renal cortical tubules and present in the glomeruli at much lower level^14^. However, the expression pattern and localization of mPGES-2 in the sepsis-induced AKI mice has not been reported yet. In this study, the widely used LPS model in sepsis research was adopted as the study subject. We first detected the expression pattern of mPGES-2 in LPS-induced AKI mice at the whole animal level. We then examined the effect of mPGES-2 knockdown at whole animal level in mice with LPS-induced AKI. Further, at the cellular level, we overexpressed and knocked down mPGES-2 in renal tubular epithelial cells by transfection of mPGES-2 overexpression plasmids and siRNA, respectively, and utilized autophagy inhibitor and inducer to analyze whether mPGES-2 exerts its roles by regulating autophagy with the hope to reveal the roles of mPGES-2 in LPS-induced AKI and its underlying mechanisms and provide new ideas and experimental clues for the prevention and treatment of AKI caused by sepsis and other factors.

## Results

### Effects of LPS on kidney function and pathological changes of mice

**Figure 1:**
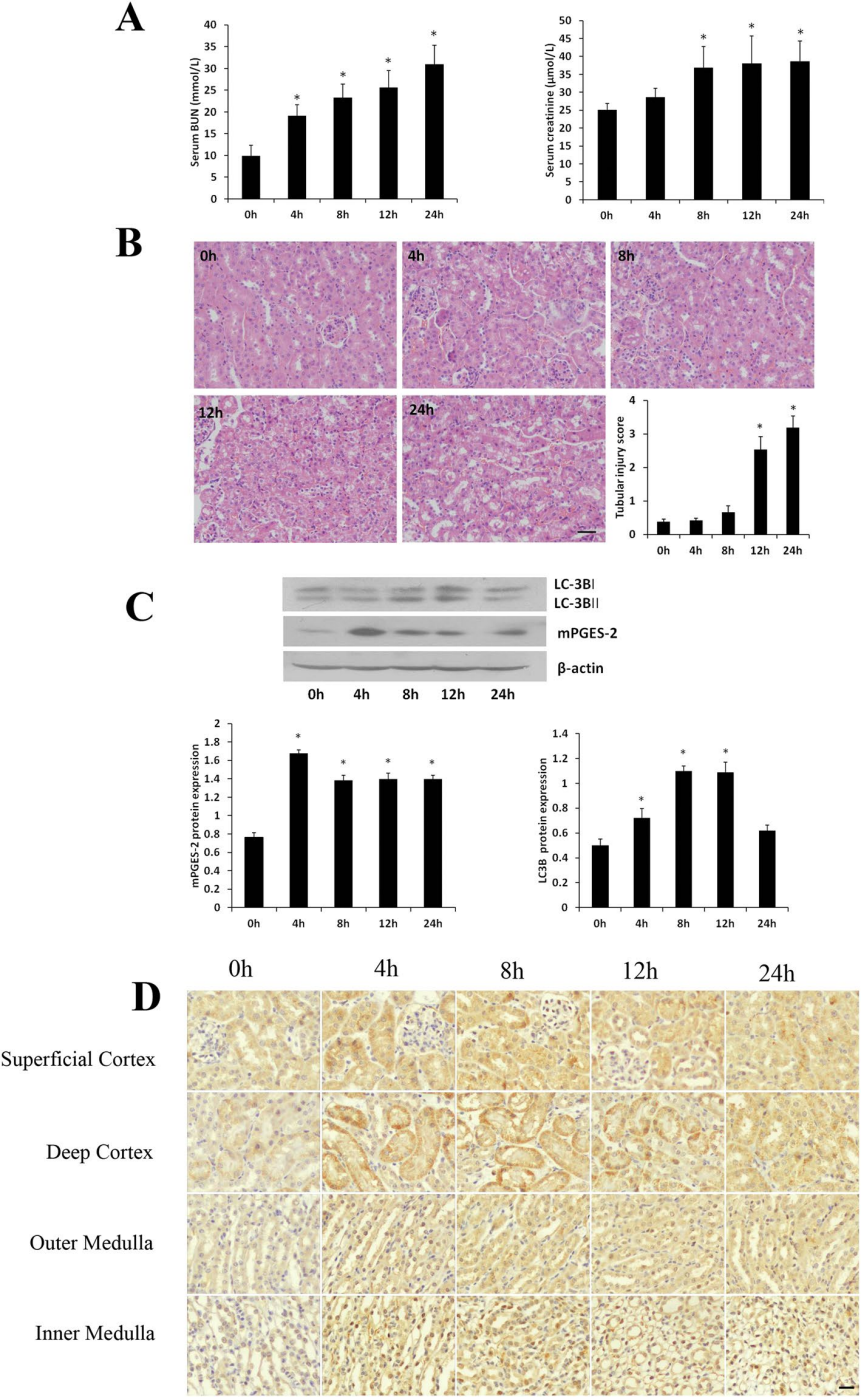
Expression of mPGES-2 in LPS-induce acute kidney injury mouse model. (**A**) Shown are the levels of serum urea nitrogen and creatinine measured at different time points. (**B**) Shown are the degrees of kidney injury and injury scores in mice at different time points after LPS treatment. Scale bar: 50 μm. (**C**) Shown are the expressions of mPGES-2 and LC-3B in kidney of mice with LPS-induce AKI. The histogram below shows the gray scale ratio of mPGES-2 to β-actin and LC3B-II/LC3B-I. (**D**) Shown are distributions of mPGES-2 in renal cortex (upper panel) and medulla (lower panel) of mice with LPS-induced AKI. Scale = 50 μm. *P < 0.05 versus the control group. Data are mean ± SD. (n = 6).

The levels of serum urea nitrogen and creatinine in mice increased gradually with the prolongation of LPS treatment, showing significant difference from those of the normal control mice (Fig. 1A). The renal tissues of mice in the control group had clear structures without degeneration, atrophy, swelling and necrosis of the renal tubular epithelial cells or inflammatory infiltration. By comparison, mice treated with LPS for 12 h and 24 h exhibited marked edema, vacuolar degeneration and luminal narrowing of the renal tubular epithelial cells (Fig. 1B).

### Expression of mPGES-2 in kidney of mice with LPS-induced AKI

Western blotting results showed that mPGES-2 protein expression is very low in the kidney of the control mice, and increased significantly after LPS treatment, reaching its peak at 4 h and maintaining at levels higher than the control at 24 h of LPS stimulation (Fig. 1C). In addition, the expression of autophagy-related protein LC3B was also examined. Usually, the ratio of LC3-II/LC3-I is used to represent the autophagic level^20, 21^. Our results showed that the ratio of LC3B-II/LC3B-I increased with the increase of LPS stimulation time, reaching the peak at 8 h, maintained at the peak to 12 h and significantly decreased at 24 h of LPS stimulation (Fig. 1C). Since mice treated with LPS for 12 h exhibited markedly damage in the kidney and the highest autophagy level, this time point was used in the subsequent experiments. In order to further clarify the location of mPGES-2 protein in the kidney, immunohistochemical method was used to detect the distribution of mPGES-2 in kidney of AKI mice. The results showed that mPGES-2 was weakly expressed in the control mice. It is mainly located in renal cortex and cytoplasm of the proximal tubule epithelial cells, but little in glomeruli and renal medulla. In addition, LPS treatment significantly increased mPGES-2 expression in tubules of renal cortex and medulla, and mPGES-2 translocation was not observed in kidney of mice during AKI (Fig. 1D).

### Infection efficiency of mPGES-2 gene 4-in-1 shRNA adenovirus and the confirmation of mPGES-2 gene knockdown in vivo

**Figure 2:**
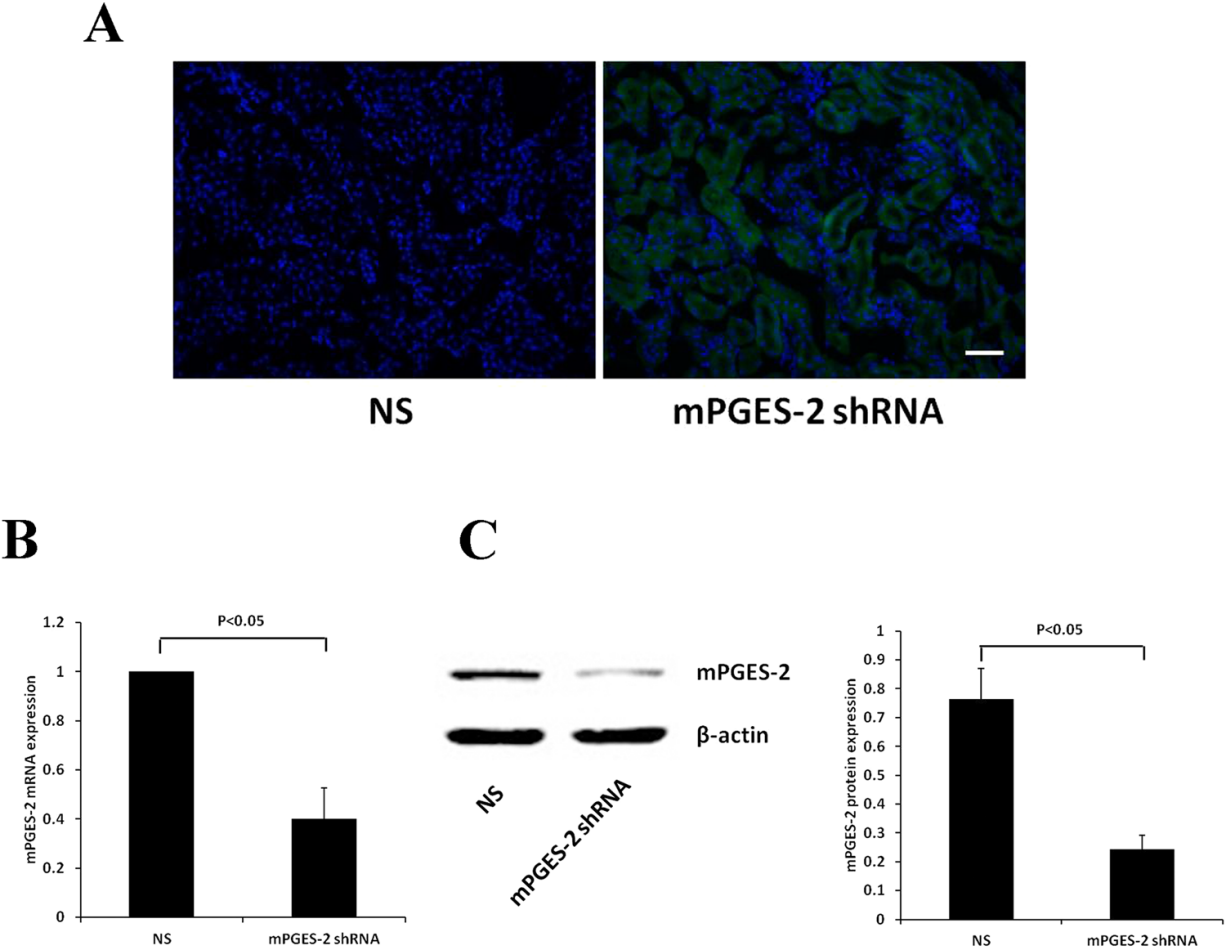
Infection efficiency of mPGES-2 gene 4-in-1 shRNA adenovirus and confirmation of mPGES-2 gene knockdown *in vivo*. mPGES-2 shRNA adenovirus with GFP or saline was administered into mice via tail vein injection. (**A**) The expression of GFP in the frozen biopsies was observed under a fluorescence microscope to determine whether the mPGES-2 shRNA is targeted to the kidney. Scale bar: 50 μm. (**B**,**C**) The mPGES-2 expression levels were detected by real-time PCR and western blot analysis in renal tissues of mPGES-2 shRNA adenovirus infected mice and control mice. Data shown are mean ± SD. (n = 3).

The successful construction of mPGES-2 gene 4-in-1 shRNA adenovirus with green fluorescent protein (GFP) gene was determined by Sanger sequencing. To demonstrate the infection efficiency of mPGES-2 shRNA adenovirus *in vivo*, frozen slices of mouse kidneys were examined using fluorescent microscopy. After six days of mPGES-2 shRNA adenovirus administration, GFP were observed in the tubular cells of mouse kidneys, suggesting that mPGES-2 shRNA targeted the renal tubular cells (Fig. 2A).

To confirm the mPGES-2 gene knockdown *in vivo*, the mRNA and protein of mPGES-2 in the kidney of mice were detected. The results showed that mPGES-2 mRNA and protein expression were significantly decreased after six days of mPGES-2 shRNA adenovirus administration compared with that of the control shRNA group (Fig. 2B,C)

### Inhibition of mPGES-2 increases mortality and renal impairment of mice with LPS-induced AKI

**Figure 3:**
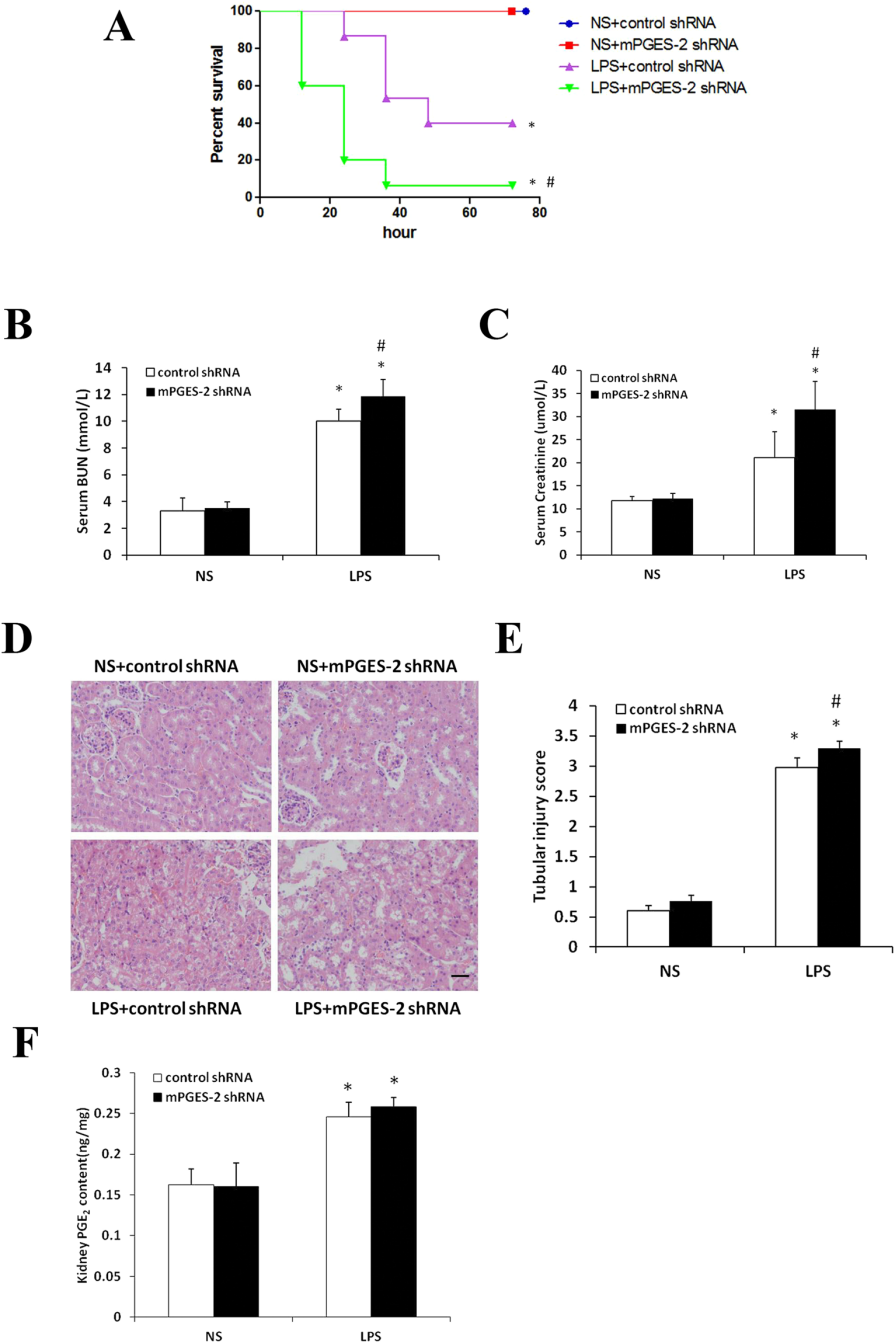
Knockdown of mPGES-2 increases the mortality and promotes renal impairment of mice with LPS-induce AKI. (**A**) Shown is the survival curve of mice with LPS-induced AKI after mPGES-2 inhibition (n = 15). (**B**) and (**C**) Shown are the levels of serum urea nitrogen and creatinine, respectively, two indices of renal function, in mice with LPS-induced AKI after mPGES-2 inhibition (n ≥ 6). (**D**) Shown are the results of HE staining, indicating kidney injury in mice with LPS-induced AKI after mPGES-2 inhibition (n ≥ 6). Scale bar: 50 μm. (**E**) Shown are kidney injury scores in mice with LPS-induced AKI after mPGES-2 inhibition (n ≥ 6). (**F**) Shown are the kidney PGE_2_ content in mice with LPS-induced AKI after mPGES-2 inhibition. *P < 0.01 versus the NS group. ^#^ *P* < 0.05 versus the LPS + control shRNA group. Data are mean ± SD.

LPS treatment of mPGES-2 shRNA adenovirus infected mice resulted in a significant reduction in 72-h survival rate from 40% in the control mice to 10% (Fig. 3A). In addition, kidney function examination showed that serum urea nitrogen and creatinine levels were significantly higher in LPS-treated mice than those in untreated mice. Particularly, these increases were more significant in mPGES-2 shRNA adenovirus infected mice than in control mice (Fig. 3B,C). HE staining showed that control and mPGES-2 shRNA adenovirus infected mice without LPS treatment had clear and integrate renal structures. After LPS treatment, the renal tubular epithelial cells underwent edema and vacuolar degeneration and these phenomena were more sever in mPGES-2 shRNA adenovirus infected mice (Fig. 3D), showing statistically significant difference in histological scores (Fig. 3E). In addition, LPS treatment increased PGE_2_ content in kidney of both control and mPGES-2 knockdown mice. However, there was no significant difference between them (Fig. 3F).

### Effects of mPGES-2 knockdown on autophagy and apoptosis in kidney of mice with LPS-induced AKI

**Figure 4:**
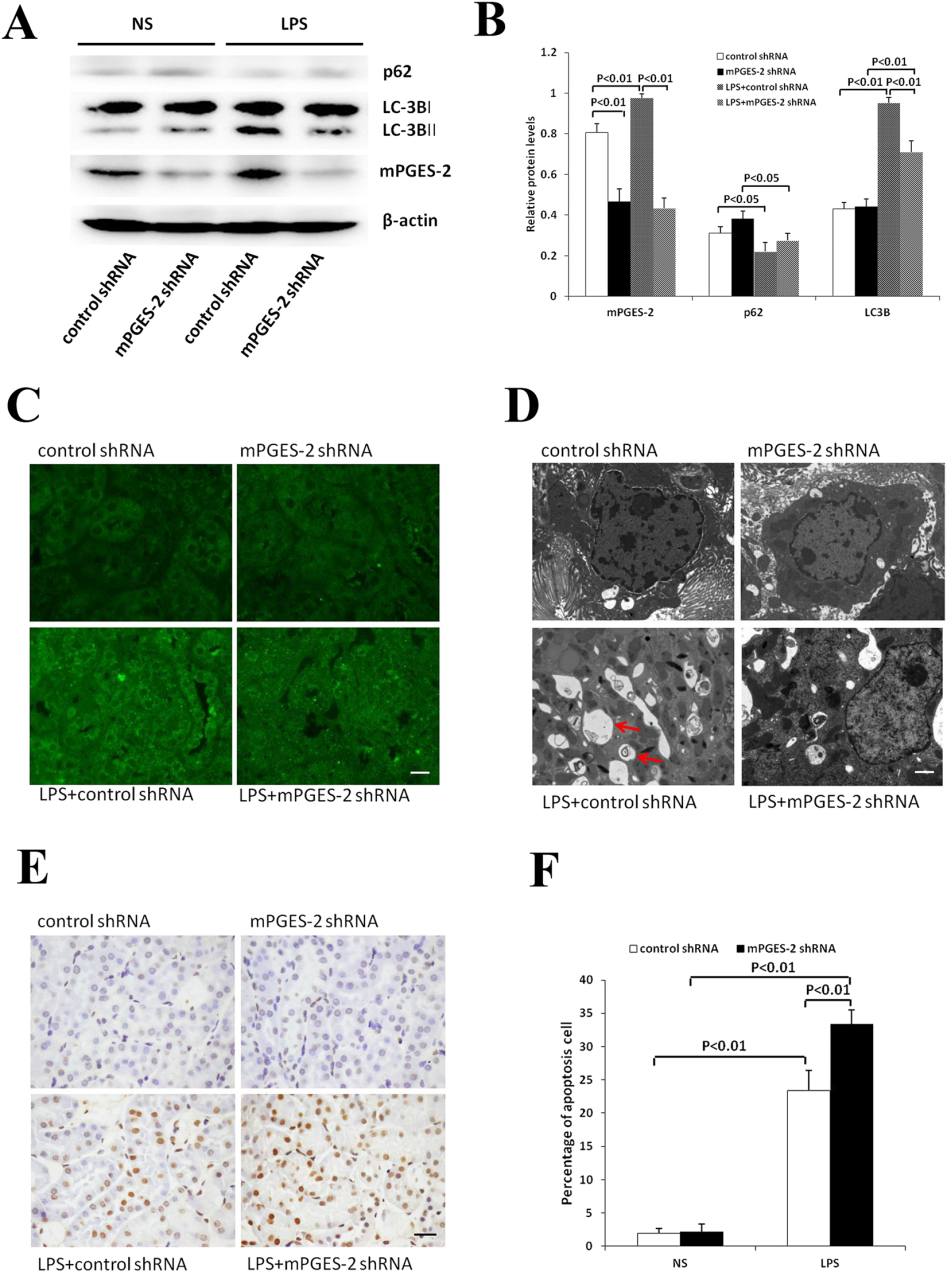
Inhibition of mPGES-2 can promote LPS-induced AKI in mice by down-regulating autophagy. (**A**) Shown are the expressions of mPGES-2, LC-3B and p62 proteins in renal tissues of mice with LPS-induced AKI after inhibition of mPGES-2. (**B**) Shown are the gray scale ratios of mPGES-2/β-actin, LC3B-II/LC3B-I and p62/β-actin. (**C**) Shown are the immunofluorescence results indicating that the expression of LC-3B in renal tissue was inhibited after mPGES-2 inhibition. Scale: 20 μm. (**D**) Shown are images obtained under electron microscope, showing that the production of autophagosomes in renal tissues of mice with LPS-induced AKI after mPGES-2 inhibition. Scale = 2 μm. Arrows indicate autophagosomes. (**E**) Shown are the apoptosis of renal tubular epithelial cells detected by TUNEL staining. Scale = 20 μm. (**F**) Shown are the percentage of apoptotic cells. Data are mean ± SD. (n ≥ 6).

The expression of mPGES-2 was detected by western blot after mPGES-2 gene was knocked down by adenovirus infection. The results showed that the expression of mPGES-2 protein in kidney decreased significantly regardless of LPS treatment, while the expression of mPGES-2 was significantly increased in LPS-treated control mice. Then, expression levels of autophagy markers LC3/Atg8 and p62/SQSTM1 were detected by western blot. P62/SQSTM1 is a multifunctional protein that acts as a selective substrate in autophagy. It binds to ubiquitinated proteins, and forms complexes with LC3, resulting in p62 transportation into autophagolysosome and degradation. On the contrary, p62 is accumulated when autophagy is defective. Thus, p62 can be used as a marker to detect autophagic activity^22, 23^. Our results showed that LPS treatment increased LC3B-II/LC3B-I ratio and decreased p62 level in both mPGES-2 downregulated and control mice (Fig. 4A,B). But the increment of LC3B-II/LC3B-I ratio in mPGES-2 shRNA adenovirus infected mice was significantly lower than that in the control mice. These results suggest that the level of autophagy in kidney is decreased after mPGES-2 downregulation. In addition, autophagy level can also be observed using immunofluorescence^20^ and transmission electron microscopy^20^. Because unbounded LC3-I is distributed in the cytoplasm and converted to LC3-II, which was localized on the autophagic membrane, after autophagy activation, under fluorescence microscopy, it is presented as bright fluorescence dots. Thus, the number of these small fluorescent dots can represent the level of intracellular autophagy. Immunofluorescence showed that autophagosome in the renal tubules was significantly increased after LPS stimulation, but this increase was significantly reduced in mPGES-2 shRNA adenovirus infected mice (Fig. 4C). Observation of typical autophagosome structure under transmission electron microscope has been the gold standard for autophagy detection^20^. Our electron microscopic results showed that LPS treatment significantly increased autophagic vacuoles in kidney. But this increase was significantly reduced in mPGES-2 shRNA adenovirus infected mice (Fig. 4D). TUNEL staining showed that LPS treatment significantly increased the number of brownish-yellow apoptotic cells in both control and mPGES-2 shRNA adenovirus infected mice. But this increase was more dramatic in mPGES-2 shRNA adenovirus infected mice than in control mice (Fig. 4E,F).

### Effects of LPS on the injury of HK2 cells

**Figure 5:**
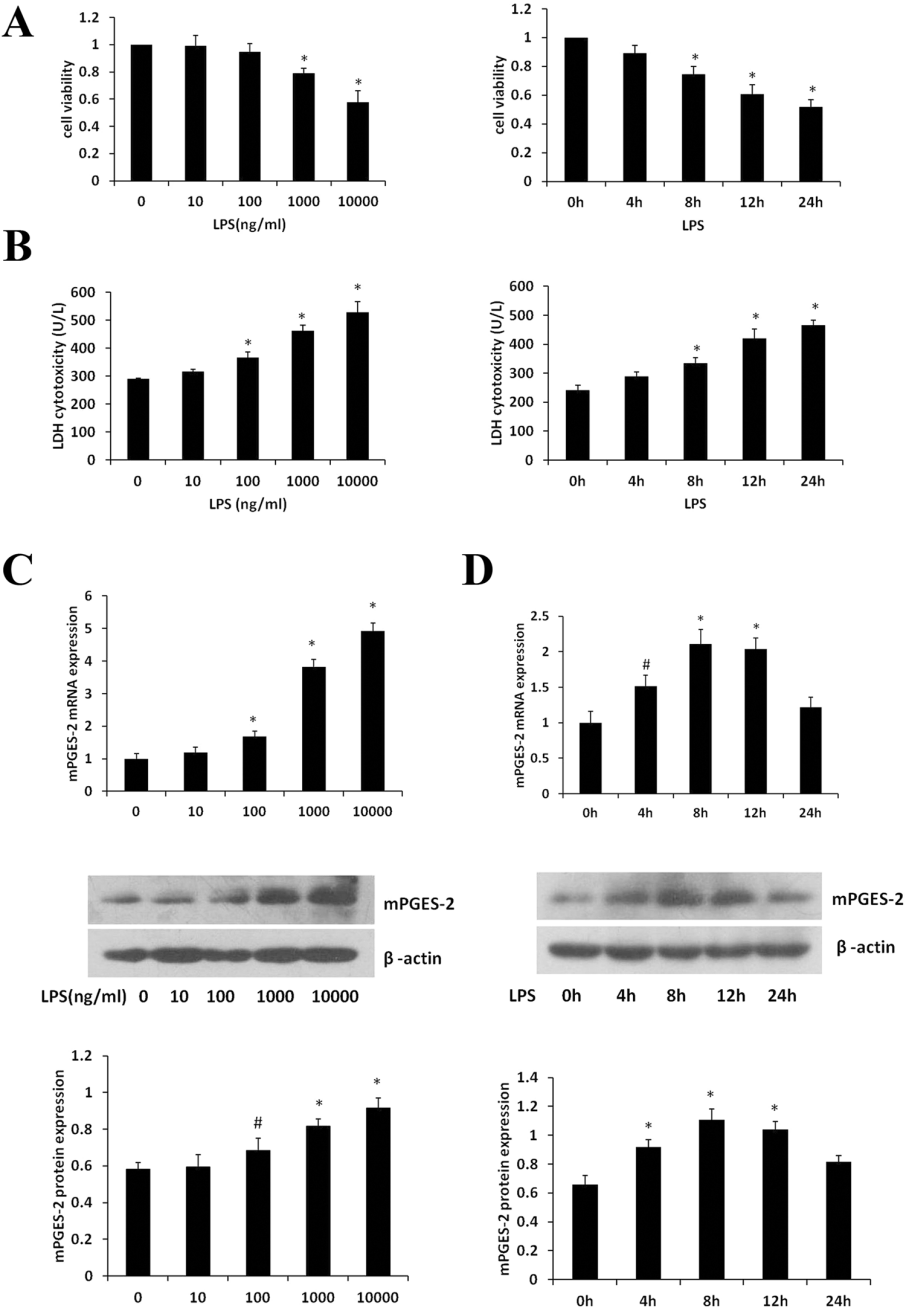
Effect of LPS on HK-2 cells injury and expression of mPGES-2 in LPS-treated HK-2 cells. (**A**) The effects of treatment with LPS at different concentrations for 24 h on HK-2 cells viability (left panel) and LDH release (right panel). (**B**) Effects of 1000 ng/ml LPS treatment for different time on the viability of HK-2 cells (left) and LDH release (right). (**C**) Effects of LPS treatment for 12 h on expression of mPGES-2 mRNA (upper panel) and mPGES-2 protein (middle panel) in HK-2 cells. The lower panel shows the gray scale ratio of mPGES-2 protein to β-actin. (**D**) The expression of mPGES-2 mRNA (upper panel) and mPGES-2 protein (middle map) in HK-2 cells after different time of LPS treatment. The lower panel shows the gray scale ratio of mPGES-2 protein to β-actin. *P < 0.01, ^#^ *P* < 0.05versus the control group. Data are mean ± SD. (n = 3).

Because immunohistochemistry showed that mPGES-2 is mainly expressed in cortical renal tubular epithelial cells, renal tubular epithelial cell line HK-2 was used to examine the effects of mPGES-2 at cellular level. First, cell viability and LDH level in the medium were measured using CCK-8 assay and LDH cytotoxicity assay after 24 h of LPS treatment with different concentrations. The results showed that LPS decreased cell viability, but increased LDH level in medium in a dose dependent manner (Fig. 5A). Next, cell viability and LDH level in the medium were measured after 1000 ng/ml LPS treatment for different times. The results showed that LPS decreased cell viability, but increased LDH level in medium in a time dependent manner (Fig. 5B).

### Expression of mPGES-2 in LPS-treated HK2 cells

First, HK-2 cells were treated with different concentrations of LPS for 12 h, and mPGES-2 mRNA and protein levels were detected. The results showed that the levels of mPGES-2 mRNA and protein increased gradually with LPS concentration increasing and reached significantly different level at the concentration of 1000 ng/ml (Fig. 5C). Therefore, this concentration was used in all experiments except those indicated. Then, HK-2 cells were treated with 1000 ng/ml LPS for different times and mPGES-2 mRNA and protein levels were examined. The results showed that the levels of mPGES-2 mRNA and protein increased after 4 h of LPS treatment, reached their peaks at 8–12 h, then gradually decreased to the level still higher than the basal level at 24 h (Fig. 5D). Therefore, cells were treated with LPS for 12 h in all experiments except those indicated.

### Effects of mPGES-2 overexpression on LPS-induced viability, autophagy and apoptosis of HK-2 cells

**Figure 6:**
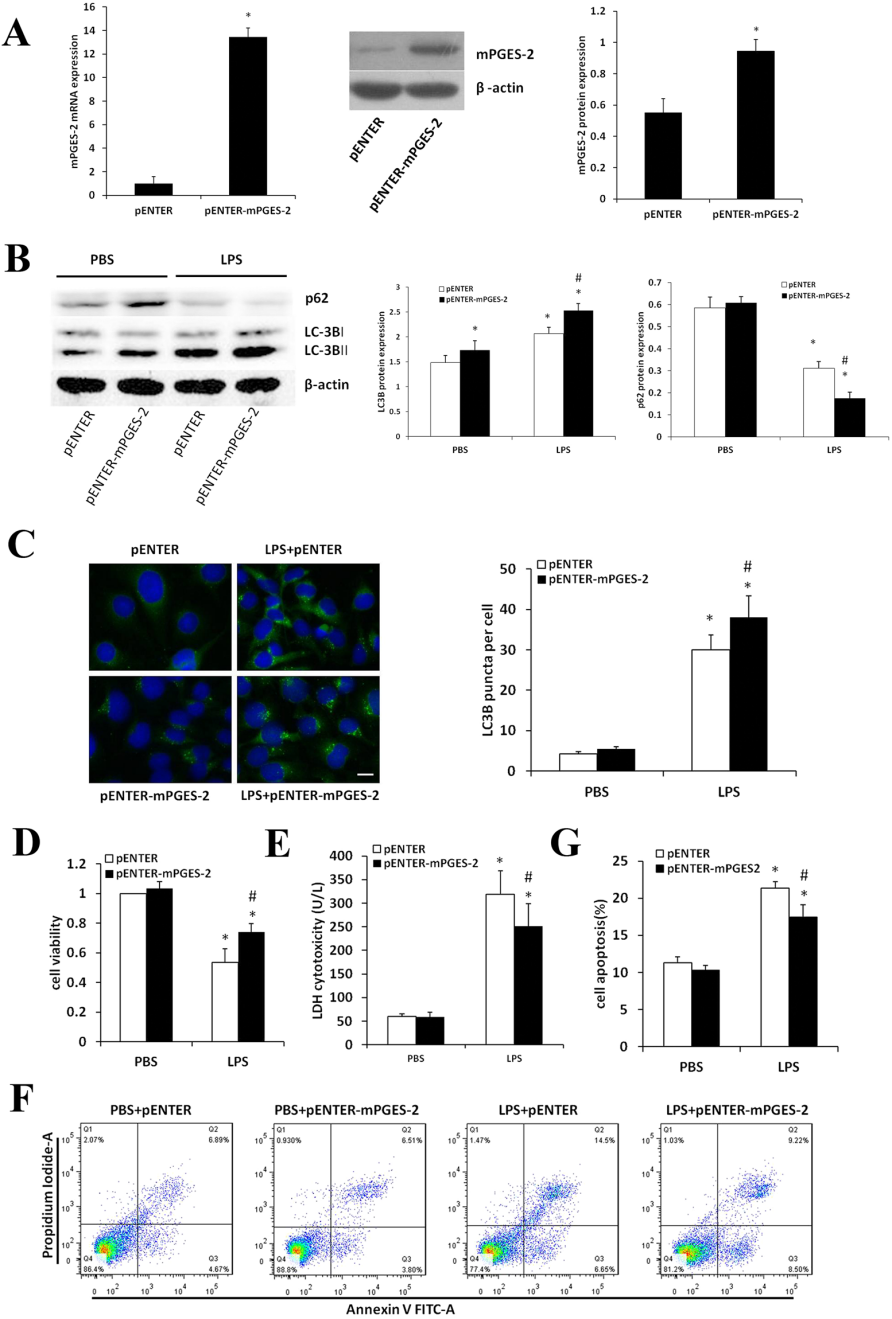
Effects of mPGES-2 overexpression on autophagy and apoptosis of HK-2 cells treated with LPS. (**A**) Shown are the ratios of mPGES-2/β-actin in HK-2 cells transfected with control plasmid (pENTER) and *mPGES-2* plasmid (pENTER-*mPGES-2*) measured by qRT-PCR (left panel) and immunoblot analyses (middle panel). Right panel shows the ratios of mPGES-2/β-actin. (**B**) Shown are immunoblot of LC3B-II/LC3B-I and p62/β-actin in HK-2 cells transfected with control plasmid (pENTER) and *mPGES-2* plasmid (pENTER-*mPGES-2*) after treatment with LPS (1000 ng/ml) for 12 h (left panel) and their ratio (right panel). (**C**) Shown are the immunofluorescence analyses of LC3B protein in HK-2 cells in different groups treated with 1000 ng/mL LPS for 12 h (left panel, scale bar: 20 μm) and the average number of autophagosomes per cell in at least 30 cells HK-2 cells (right panel). (**D**) Shown are cells viability of different groups measured using CCK8 analysis. (**E**) Shown are the LDH cytotoxicity in different groups. (**F**) Shown are the apoptosis of HK-2 cells in different groups examined by flow cytometry. (**G**) Shown are the ratios of cell apoptosis. *P < 0.05 versus the control group. ^#^ *P < *0.05 versus the LPS + control plasmid group. Data are mean ± SD. (n = 3).

To investigate the effect of mPGES-2 on LPS-induced viability, autophagy and apoptosis of renal tubular epithelial cells, HK-2 cells were transiently transfected with control and mPGES-2 plasmid, and expression of mPGES-2 was verified by both western blot and qRT-PCR. The results showed the levels of mPGES-2 mRNA and protein were significantly increased in HK-2 cells transfected with mPGES-2 plasmid (Fig. 6A). In addition, these cells were treated with LPS to explore the effects of mPGES-2 on LPS-induced viability, autophagy and apoptosis of HK-2 cells. The results showed that mPGES-2 overexpression significantly increased the ratio of LC3B-II/LC3B-I, but decreased the expression of p62 after LPS treatment (Fig. 6B). Similarly, immunofluorescence showed that mPGES-2 overexpression significantly increased the number of autophagosomes after LPS stimulation (Fig. 6C). In addition, mPGES-2 overexpression significantly increased cell viability and decreased LDH release (Fig. 6D,E). Flow cytometry showed that mPGES-2 overexpression significantly reduced apoptosis of HK-2 cells (Fig. 6F,G).

### Effects of interfering mPGES-2 expression on LPS-induced viability, autophagy and apoptosis of HK-2 cells

**Figure 7:**
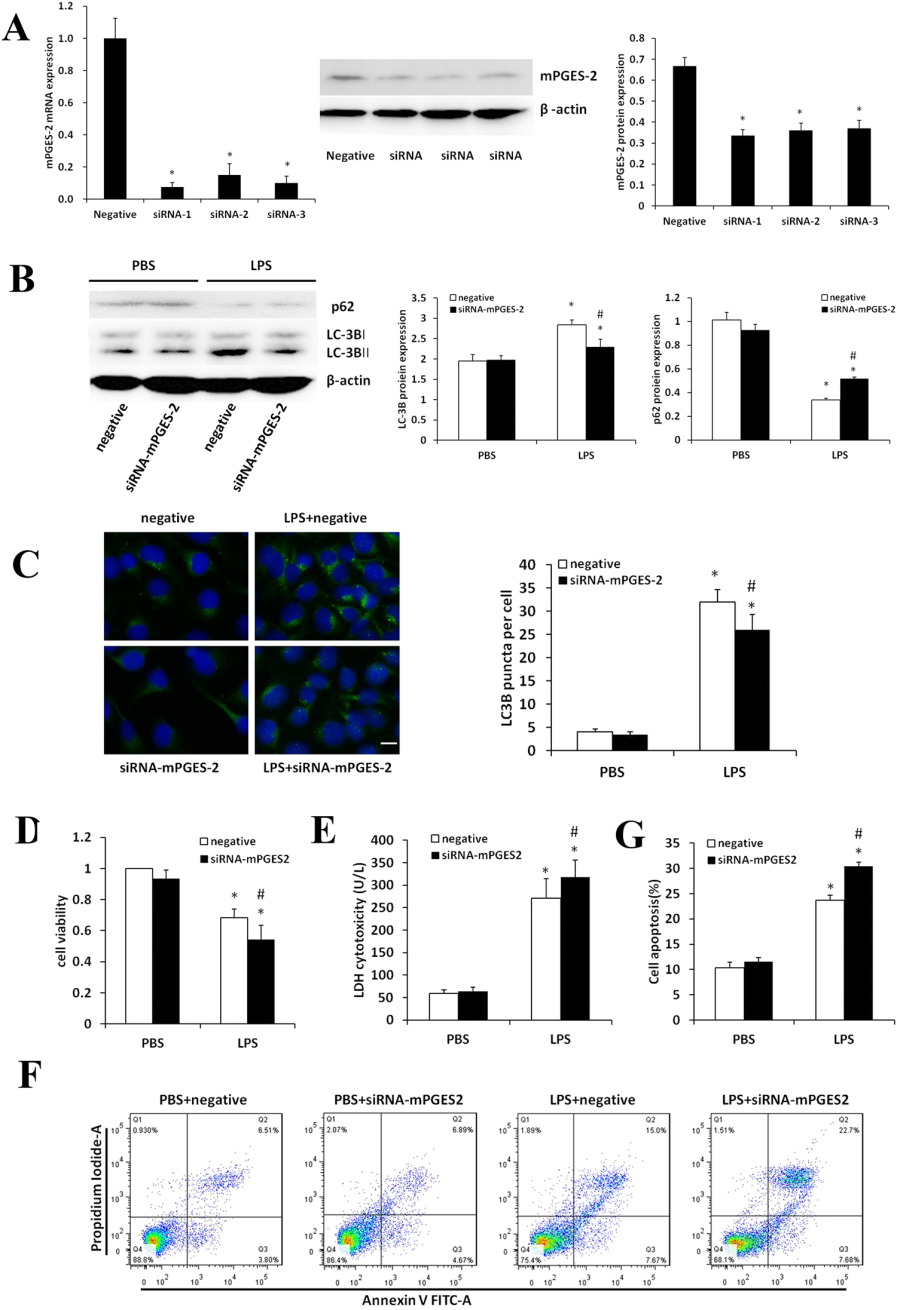
Effects of mPGES-2 interference on autophagy and apoptosis of HK-2 cells treated with LPS. (**A**) Shown are the levels of mPGES-2 expression in HK-2 cells transfected with control siRNA and siRNA-*mPGES-2* after treatment with 1000 ng/ml LPS for 12 h measured by qRT-PCR (left panel) and immunoblot analyses (middle panel). Right panel shows the ratios of mPGES-2/β-actin. (**B**) Shown are the immunoblot results of LC-3B and p62 (left panel) and the ratios of LC3B-II/LC3B-I and p62/β-actin (right panel) in HK-2 cells transfected with control siRNA and siRNA-*mPGES-2* after treatment with 1000 ng/ml LPS for 12 h. (**C**) Shown are the immunofluorescence analyses of HK-2 cells in different groups treated with 1000 ng/mL LPS for 12 h (left panel, scale bar: 20 μm) and the average number of autophagosomes per cell measured in at least 30 cells (right panel). (**D**) Shown are the viabilities of HK-2 cells in different groups measured using CCK8 analysis. (**E**) Shown are the LDH cytotoxicity detection in different groups. (**F**) Shown are the apoptosis of HK-2 cells in different groups examined by flow cytometry. (**G**) Shown are the ratios of apoptotic cells in different groups (right panel). *P < 0.05 versus the control group. ^#^ *P* < 0.05 versus the LPS + control plasmid group. Data are mean ± SD. (n = 3).

The expression of mPGES-2 was significantly inhibited in HK-2 cells transfected with mPGES-2 siRNA at both mRNA and protein levels (Fig. 7A). In contrast to mPGES-2 overexpressed HK-2 cells, mPGES-2 downregulation significantly reduced the ratio of LC3B-II/LC3B-I (Fig. 7B). Immunofluorescence showed that mPGES-2 downregulation significantly reduced the number of autophagosomes after LPS stimulation (Fig. 7C). Moreover, mPGES-2 downregulation significantly decreased cell viability, but increased LDH release (Fig. 7D,E). Flow cytometry showed that mPGES-2 downregulation significantly increased apoptosis of HK-2 cells induced by LPS (Fig. 7F,G). These results suggest that mPGES-2 can promote autophagy, inhibit apoptosis and promote proliferation of renal tubular epithelial cells, and inhibit the release of toxic substance LDH.

### Effects of 3-MA and rapamycin on LPS-induced autophagy and apoptosis of HK-2 cells

**Figure 8:**
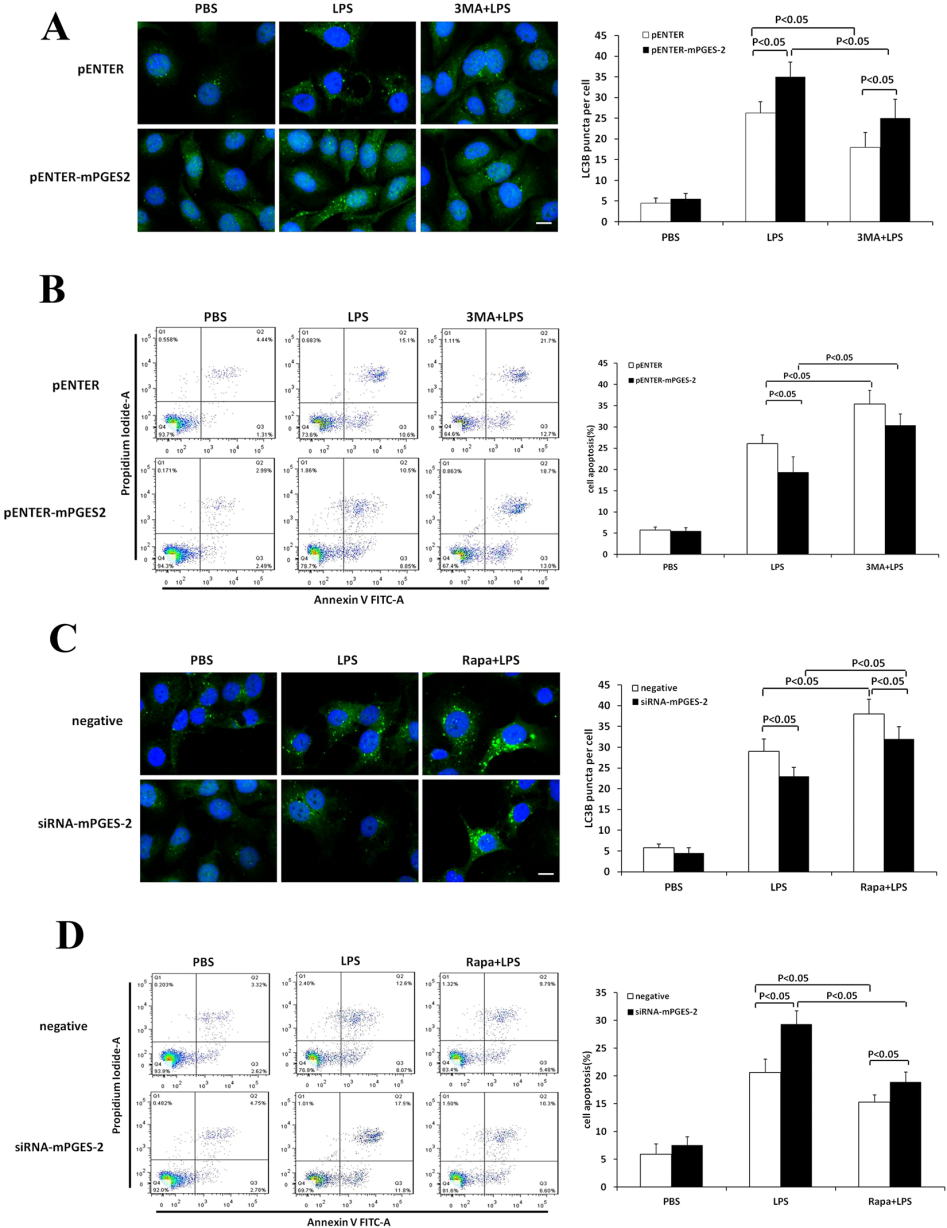
Effects of 3-MA and rapamycin treatments on autophagy and apoptosis of HK-2 cells treated with LPS. (**A**) Shown are the immunofluorescence analyses of mPGES-2 overexpressing and control HK-2 cells after treatment 5 mM 3-MA and 1000 ng/ml LPS for 12 h (left panel, scale bar: 20 μm) and the average number of autophagosomes per cell measured in at least 30 cells (right panel). (**B**) Shown are the apoptosis of mPGES-2 overexpressing and control HK-2 cells after treatment 5 mM 3-MA and 1000 ng/ml LPS for 12 h measured using flow cytometry and the ratios of cell apoptosis (right panel). (**C**) Shown are the immunofluorescence analyses of mPGES-2 interfering and control HK-2 cells after treatment 50 nM rapamycin and 1000 ng/ml LPS for 12 h (left panel, scale bar: 20 μm) and the average number of autophagosomes per cell measured in at least 30 cells (right panel). (**D**) Shown are the apoptosis of mPGES-2 interfering and control HK-2 cells after treatment 50 nM rapamycin and 1000 ng/ml LPS for 12 h measured using flow cytometry and the ratios of cell apoptosis (right panel). Data are mean ± SD. (n = 3).

**Figure 9:**
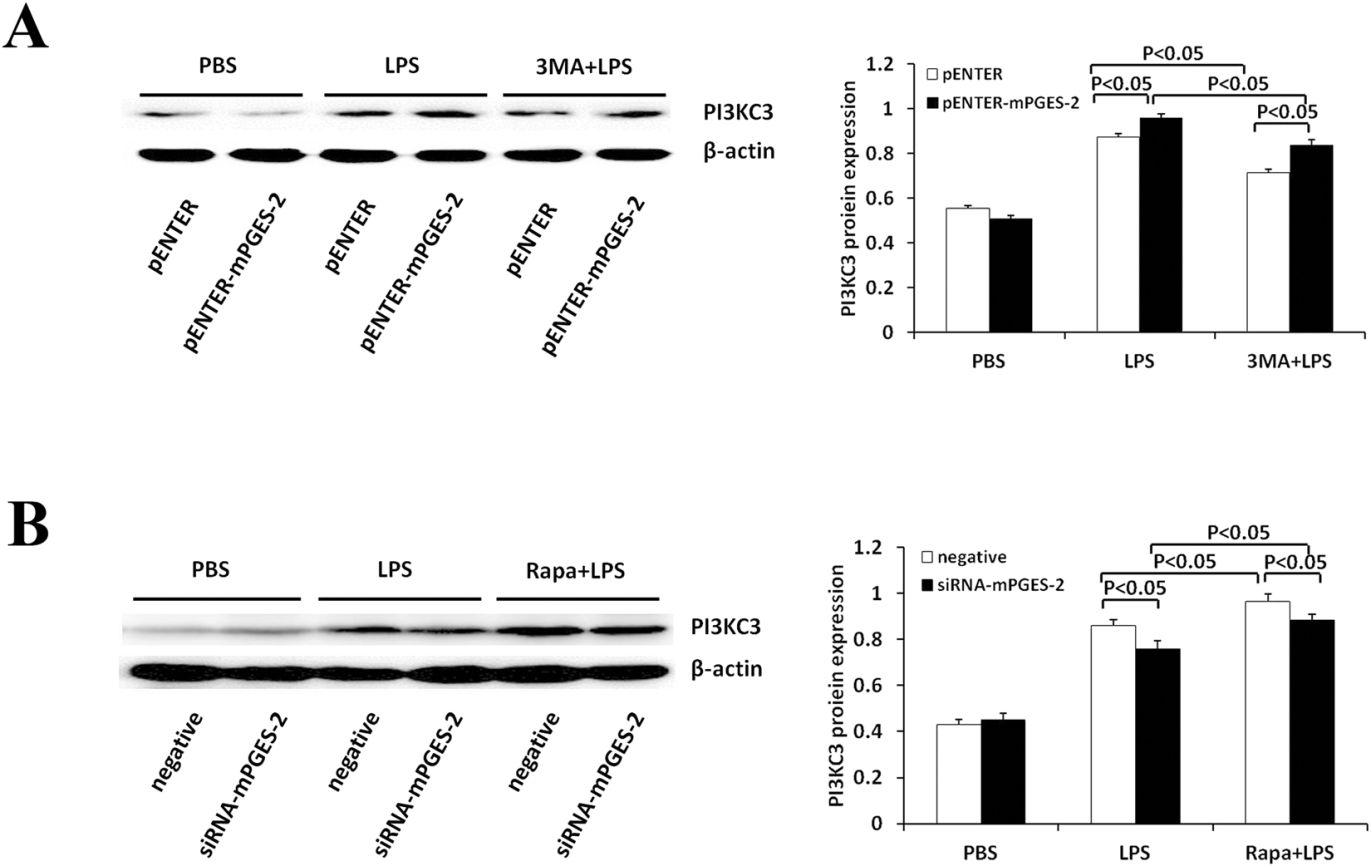
Effects of 3-MA and rapamycin treatments on expression of PI3KC3 protein in HK-2 cells treated with LPS. (**A**) Shown are the immunoblot analyses of mPGES-2 overexpressing and control HK-2 cells after treatment with 5 mM 3-MA and 1000 ng/ml LPS for 12 h (left panel) and the gray scale ratios of PI3KC3/β-actin (right panel). (**B**) Shown are the immunoblot analyses of mPGES-2 interfering and control HK-2 cells after treatment with 50 nM rapamycin and 1000 ng/ml LPS for 12 h (left panel) and the gray scale ratios of PI3KC3/β-actin (right panel). Data shown are mean ± SD. (n = 3).

To further clarify that mPGES-2 exerts its protective effect on renal tubular epithelial cells through regulating autophagy, mPGES-2 overexpressed HK-2 cells were treated with autophagy inhibitor 3-MA, and mPGES-2 downregulated HK-2 cells were treated with autophagy-inducer rapamycin. The results showed that 3-MA treatment significantly decreased LPS-induced autophagy (Fig. 8A), but increased LPS-induced apoptosis in both control and mPGES-2 overexpressed HK-2 cells (Fig. 8B). In contrast, rapamycin treatment significantly increased LPS-induced autophagy (Fig. 8C), but decreased the ratio of apoptosis (Fig. 8D) in both control and mPGES-2 downregulated HK-2 cells. In addition, we found that treatment of 3-MA significantly decreased mPGES-2-induced expression of PI3KC3 protein, while treatment of rapamycin, an autophagy inducer, further increased LPS-induced the expression of PI3KC3 protein (Fig. 9A,B).

### Role of mPGES-2 in H2O2-induced HK-2 injury model

To clarify whether the kidney protective effect of mPGES-2 is universal under different pathological conditions, we established another injury model of HK-2 cells using H_2_O_2_. Consistent with the LPS model, H_2_O_2_ treatment induced the expression of mPGES-2 and enhanced autophagy (Supplementary Fig. 1A–C). mPGES-2 overexpression significantly increased the ratio of LC3B-II/LC3B-I and reduced apoptosis of HK-2 cells. On the contrary, mPGES-2 interference significantly reduced the ratio of LC3B-II/LC3B-I and promoted apoptosis of HK-2 cells (Supplementary Fig. 1D–G). Our results suggest that mPGES-2 also ameliorates H_2_O_2_ induced damage by regulating autophagy of HK-2 cells.

## Discussion

At present, the biological functions of mPGES-2 *in vivo* are not clear. This study for the first time explored the expression and localization of mPGES-2 in endotoxemia mouse model. We found LPS could induce mPGES-2 expression and up-regulation of mPGES-2 can protect renal tubular epithelial cells by promoting autophagy and inhibiting apoptosis.

The occurrence of sepsis-induced AKI is affected by many factors. In addition to hemodynamic changes, other factors such as renal cell apoptosis, endotoxin-induced complex inflammation and immune network response, endothelial dysfunction, glomerular embolization and necrosis-induced renal tubular obstruction are all involved in its pathophysiological changes^24–26^. Among them, apoptosis of renal tubular epithelial cells may play an important role in the development of sepsis induced AKI^27^. The use of caspase inhibitors can not only prevent renal cell apoptosis, but also inhibit the renal tissue inflammatory response, thereby protecting LPS-induced kidney damages^28, 29^. Our study showed that inhibiting mPGES-2 expression significantly increased the apoptosis rate of renal tubular epithelial cells, suggesting that mPGES-2 may play a protective role by inhibiting renal tubular cell apoptosis. We also found that inhibiting mPGES-2 expression decreased renal autophagy. As a cellular stress response, autophagy plays important roles in many diseases^30–32^. Increased autophagy has been observed in AKI due to various causes (e.g., ischemia-reperfusion injury, cisplatin, LPS, etc.), while knockout of autophagy-related genes aggravated kidney damages^8, 33, 34^. Our results also showed that decrease in autophagy in LPS-induced AKI resulted in more severe renal injury in mice. The balance of autophagy and apoptosis in the body is affected by a variety of factors and has important impacts on the prognosis of the disease. For example, enhancing macroautophagy inhibits myocardial cell apoptosis and protects against ischemia/reperfusion injury in cardiac myocytes^35, 36^. Autophagy induction protect against cisplatin-stimulated tubular cell apoptosis^37^. Enhanced autophagy by rapamycin exerts a renoprotective role via inhibiting tubular cell apoptosis during renal I/R injury^38^. Usually, the dynamic balance of autophagy and apoptosis, to a certain extent, determines the survival or death of cells. Therefore, it is important to find a common upstream factor that regulates the autophagy and apoptosis. Our *in vitro* results showed that in LPS-induced AKI model, mPGES-2 is involved in the regulation of autophagy and apoptosis in renal tubular epithelial cell, and can be a common upstream factor of them. It is therefore reasonable to speculate that mPGES-2 decreases the apoptosis rate of LPS-induced renal tubular epithelial cell by regulating autophagy activity.

To further demonstrate this hypothesis, we treated HK-2 cells with autophagy inhibitor 3-methyladenine (3-MA) and autophagy agonist rapamycin. The results showed that 3-MA treatment blocked autophagy induced by mPGES-2, and increased apoptosis rate of HK-2 cells. Previous studies have shown that 3-MA can block autophagy by inhibiting class III phosphoinositide 3-phosphate kinase (PI3KC3)^39^. The activity of PI3KC3 is necessary for the nucleation and assembly of membrane pool formation in early autophagy^20, 40^. Combined with our results, we believe that mPGES-2 can inhibit apoptosis by increasing autophagy and may play a role in the upstream of PI3KC3. In contrast, rapamycin treatment re-induced autophagy after interfering mPGES-2 expression, and reduced apoptosis rate. Rapamycin is an immunosuppressant and a negative regulator specific to mTOR signaling pathway^41, 42^. A large number of studies have shown that rapamycin can induce autophagy^43^. Our results also showed that rapamycin reversed inhibition of autophagy due to interfering mPGES-2 expression, strongly suggesting that autophagy and apoptosis induced by mPGES-2 have an upstream and downstream relationship and both of them are involved in protection of LPS-induced injury in HK-2 cells. Meanwhile, our results showed that the PI3KC3 complex was regulated by mPGES-2, which further suggested that PI3KC3 complex and/or its upstream regulatory molecules may be the targets of mPGES-2. But how mPGES-2 affects PI3KC3 pathways need to be further investigated.

To investigate whether mPGES-2 exerts protective role in other model, we stimulated HK-2 cells using H_2_O_2_. It is well known that H_2_O_2_ induces cell apoptosis^44^. Our results revealed mPGES-2 also ameliorates H_2_O_2_ induced apoptosis by regulating autophagy. These results support the nonspecific role of mPGES-2 in LPS or H_2_O_2_ induced HK-2 cell injury models.

In conclusion, mPGES-2 down-regulation aggravates acute kidney injury by inhibiting autophagy and promoting apoptosis in mice. The protective effect of mPGES-2 is mediated, at least partially, through promoting autophagy and inhibiting apoptosis of renal tubular epithelial cells.

## Materials and Methods

This study was approved by the Ethical Committee of the Animal Experimental Institute of Central South University, Changsha, China, and carried out in accordance with the approved protocol.

### Animals and preparation of LPS-induced AKI mouse model

A total of 130 male C57BL/6 mice, weighing 20–25 g, from Hunan SJA Laboratory Animal Co., Ltd. were used in the experiments. They were maintained in a clean and quiet environment with relative humidity of 40~60% and light/dark cycle of 12 h/12 h at 25 °C. Among them, 30 mice were divided into 5 groups with 6 mice in each group (n = 6) and intraperitoneally injected LPS at 10 mg/kg body weight for 0 (control), 4, 8, 12 and 24 h, respectively. Under anesthesia, their eyeballs were removed for collection of blood in a clean centrifuge tube. Subsequently, mice were scarified and their left kidneys were collected for qRT-PCR, western blot and immunohistochemistry. The other 100 mice were used to establish mPGES-2 knockout mouse model as described in Section below.

### Renal function testing and biochemical assays

Blood samples taken from the animals were placed still at room temperature for 2 h and sera were collected by centrifugation at 1000 g for 15 min. The renal function indexes such as urea nitrogen and creatinine were measured using automatic biochemical analyzer (Hitachi Modular System, Hitachi, Tokyo, Japan). Concentration of kidney PGE_2_ was determined by enzyme immunoassay (Cusabio Life science, Hubei, China).

### Observation of kidney damages by hematoxylin and eosin staining

The fresh mouse kidney tissues were fixed in 4% paraformaldehyde solution, stained hematoxylin and eosin and observed under a microscope. The degree of kidney damages was assessed using the blind method. Samples with normal kidney, renal tubular injury area <25%, 25–50%, 50–75% and 75–100% were scored 0, 1, 2, 3, and 4, respectively^45^.

### Immunohistochemistry

The expression of mPGES-2 in kidney was detected by immunohistochemistry using anti-mPGES-2 antibody at 1:100 dilution (Cayman chemical) and EnVision (TM) FLEX Mini kit according to the instructions provided by the manufacturer (Dako, K8024).

### Construction of mPGES-2 shRNA expression vectors and recombinant adenovirus

Four shRNA targeting to mPGES-2 were designed, inserted into a 4-in-1-shRNA vector with green fluorescent protein (GFP) and packed into recombinant adenovirus by Vigene Biosciences, Inc. (Ptges2-shRNA1:GCCCAGTTCTCTGCTTCTGATATTCAAGAGATATCAGAAGCAGAGAACTGGGTTTTTT,Ptges2-shRNA2:GGCTCAATGACTCCTCTGTCATTTCAAGAGAATGACAGAGGAGTCATTGAGCTTTTTT,Ptges2-shRNA3:GCCTCTATGAAGCAGCCAACAATTCAAGAGATTGTTGGCTGCTTCATAGAGGTTTTTT,Ptges2-shRNA4:GCGTGAGAAGGACTGAGATCAATTCAAGAGATTGATCTCAGTCCTTCTCACGTTTTTT). The virus was further amplified, purified, and adjusted to titer of 1.0 × 10^11^ plaque-forming units (PFU)/ml by Vigene Biosciences, Inc. Control virus and shRNA expressing virus were diluted 4-fold using normal saline (NS) as working solution. The infection efficiency was detected by fluorescence microscopy. The silencing efficiency of mPGES-2 gene and protein was determined by real time PCR and western blot analysis.

### Establishment of mPGES-2 knockout mouse model and LPS treatment

A total of 100 mice were randomly divided into four groups, namely (1) NS + control shRNA group, (2) NS + mPGES-2 shRNA group, (3) LPS + control shRNA group and (4) LPS + mPGES-2 shRNA group, with 25 mice per group. Mice were anesthetized with 4% chloral hydrate and injected 100 μL of control viral working solution or shRNA expressing viral working solution per mouse through tail vein based on their grouping. Six days after injection, mice in LPS groups were intraperitoneally injected LPS at 10 mg/kg body weight. Among them, 15 mice in each group were used to observe survival rate every 12 h until 72 h. The survival rates were used to draw the survival curve. The other 10 mice in each group were sacrificed under anesthesia at 12 h treatment with LPS or NS and their blood and tissues were taken for further analyses. Mice died within 12 h were excluded from the experiment.

### Transmission Electron Microscopy

Fresh kidneys were quickly emerged into fixative for electron microscope at 4 °C for 2–4 h. After that, samples were transferred to Wuhan Goodbio technology Co., Ltd, prepared as specimens, examined and photographed under a transmission electron microscope.

### Detection of renal tubular cell apoptosis using TUNEL method

The apoptotic level of kidney in paraffin sections was examined using TUNEL apoptosis detection kit (Roche) according to instructions provided by the manufacturer. The percentage of apoptosis cell was calculated as the number of apoptotic cells/total number of cells × 100% in 5–10 randomly selected fields.

### Cell culture

The HK-2 cell line from ATCC was maintained in DMEM/F12 medium supplemented with 10% fetal bovine serum at 37 °C in an incubator supplemented with 5% CO_2_. Medium was renewed every 2 days.

### Real time PCR

The total RNA was extracted from kidneys and HK-2 cells with TRIzol. mRNA were reverse transcribed to cDNA using PrimeScript™ RT Master Mix (Takara, Japan). Real-time PCR was performed using cDNA as template and One Step SYBR ® PrimeScript ™ RT-PCR Kit on the Biosystems 7500 Real Time PCR System. The amplification was carried out for 40 cycles at conditions of 95 °C for 30 s, 95 °C for 5 s and 60 °C for 34 s. The primer set 5′-CAT TGC TGA CAG GAT GCA GAA GG-3′ and 5′-TGC TGG AAG GTG GAC AGT GAG G-3′ as well as the primer set 5′-GGT AGA CCT CTA TGA AGC AGC C-3′ and 5′-CAT CAC TCG CAG CAC ACC ATA C-3′ were used for amplification of mouse β-actin and mPGES-2, respectively. The primer set 5′-CAC CAT TGG CAA TGA GCG GTT C-3′ and 5′-AGG TCT TTG CGG ATG TCC ACG T-3′ as well as the primer set 5′-CCT CTA TGA GGC TGC TGA CAA G-3′ and 5′-ATC ACA CGC AGC ACG CCA TAC A-3′ were used for amplification of human β-actin and mPGES-2, respectively.

### Western blot

Mouse kidney and HK-2 cells were lysed using RIPA buffer plus PMSF. Proteins in the extractions were determined using BCA method. The same amount of proteins were mixed with the same volume of 2 × SDS loading buffer, boiled at 100 °C for 10 min, subjected to electrophoresis and transferred onto membranes. The membranes were blocked at room temperature for 1 h, and then incubated overnight with primary antibodies against mPGES-2 (1:1,000 dilution, cayman chemical), microtubule-associated protein light chain 3 (LC-3B) (1:1,000 dilution, CST), p62 (1:1,000 dilution, CST), PI3KC3 (1:1000 dilution, CST) and β-actin (1:2,000 dilution, Sigma-Aldrich), respectively, at 4 °C. After washed with PBS, the membranes were then incubated with corresponding secondary antibodies for 1 h at room temperature. Signals were visualized using ECL substrate and quantified using ImageJ software.

### Immunofluorescence analyses

Paraffin sections were subjected to antigen repair, blocked using sheep serum for 30 min, and incubated with primary antibody against LC-3B (1:100, CST) overnight at 4 °C. After washed with PBS, the sections were incubated with FITC-labeled anti-rabbit secondary antibody (1:400 dilution, MultiSciences) at room temperature in the dark for 1 h, counterstained with DAPI, and photographed under a fluorescence microscope.

HK-2 cells were seeded on coverslips. After transfection and stimulation according to the experimental requirements, cells were washed with PBS three times, fixed with 100% methanol for 5 min, washed three times with PBS again, and blocked using sheep serum for 30 min. After that cells were stained with LC-3B antibody, FITC-labeled anti-rabbit secondary antibody and subjected to Immunofluorescence analyses as described above.

### Transfection of plasmids and siRNA into HK-2 cells

pENTER eukaryotic plasmids expressing mPGES-2 and small interfering RNA (siRNA) of mPGES-2 were purchased from ViGene Biosciences and Shanghai GenePharma Co., Ltd, respectively. The plasmid was extracted using Plasmid Maxprep Kit (Vigorous Biotechnology) according to the manufacturer’s instruction. Plasmid and siRNA transfections into HK-2 cells were carried out using Lipofectamine 3000™ (Invitrogen) according to the manufacturer’s instruction.

### Analysis of cell proliferation and detection of LDH level in culture supernatant

The viability of cells cultured in 96-well plates in each group was measured using CCK-8 kit (Dojindo, Japan) following the manufacturer’s instruction. The culture supernatants were used to measure LDH level using LDH cytotoxicity test kit (Nanjing Jiancheng Bioengineering Institute) following the manufacturer’s instructions.

### Apoptosis detection using flow cytometry

The cells in each group were washed, digested and subjected to apoptosis analysis using Annexin V-FITC/PI Apoptosis Detection Kit (BD Pharmingen, CA, USA) according to the manufacturer’s instructions.

### Statistical analysis

GraphPad Prism version 5.02 (GraphPad Prism Software Inc, San Diego, CA) was used for data analysis. All the experiments were repeated at least three times. The quantitative data were presented as Mean ± SD. Multiple comparisons were analyzed for significant differences by using the one-way ANOVA with Tukey post hoc test. Kaplane-Meier plots were used to illustrate survival of mice between different groups, and statistical assessment was performed by the log-rank test. A value of *P* < 0.05 was considered statistically significant.

## Electronic supplementary material

Supplementary information
