# Author Correction: Aggravation of acute kidney injury by mPGES-2 down regulation is associated with autophagy inhibition and enhanced apoptosis

**DOI:** 10.1038/s41598-018-35440-1

**Published:** 2018-11-16

**Authors:** Ting Li, Ying Liu, Jie Zhao, Shuying Miao, Yunfei Xu, Ke Liu, Meidong Liu, Guiliang Wang, Xianzhong Xiao

**Affiliations:** 1 Department of Pathophysiology, Xiangya School of Medicine, Central South University, Changsha, 410078 China; 2 Department of Physiology, Changzhi Medical College, Changzhi, 046000 China; 3 Department of Neurosurgery, Xiangya Hospital, Central South University, Changsha, 410078 China; 4 Department of Digestive Internal Medicine, Gannan Medical University Pingxiang Hospital, Pingxiang, 337055 China

Correction to: *Scientific Reports* 10.1038/s41598-017-10271-8, published online 31 August 2017

This Article contains errors.

In Figure 7 the labelling of the immunoblot analysis in panel A is incorrect, and in panel F the flow cytometry plots for ‘PBS + negative’ and ‘PBS + siRNA-mPGES2’ are incorrect. The correct Figure 7 appears below as Figure 1.

**Figure 1:**
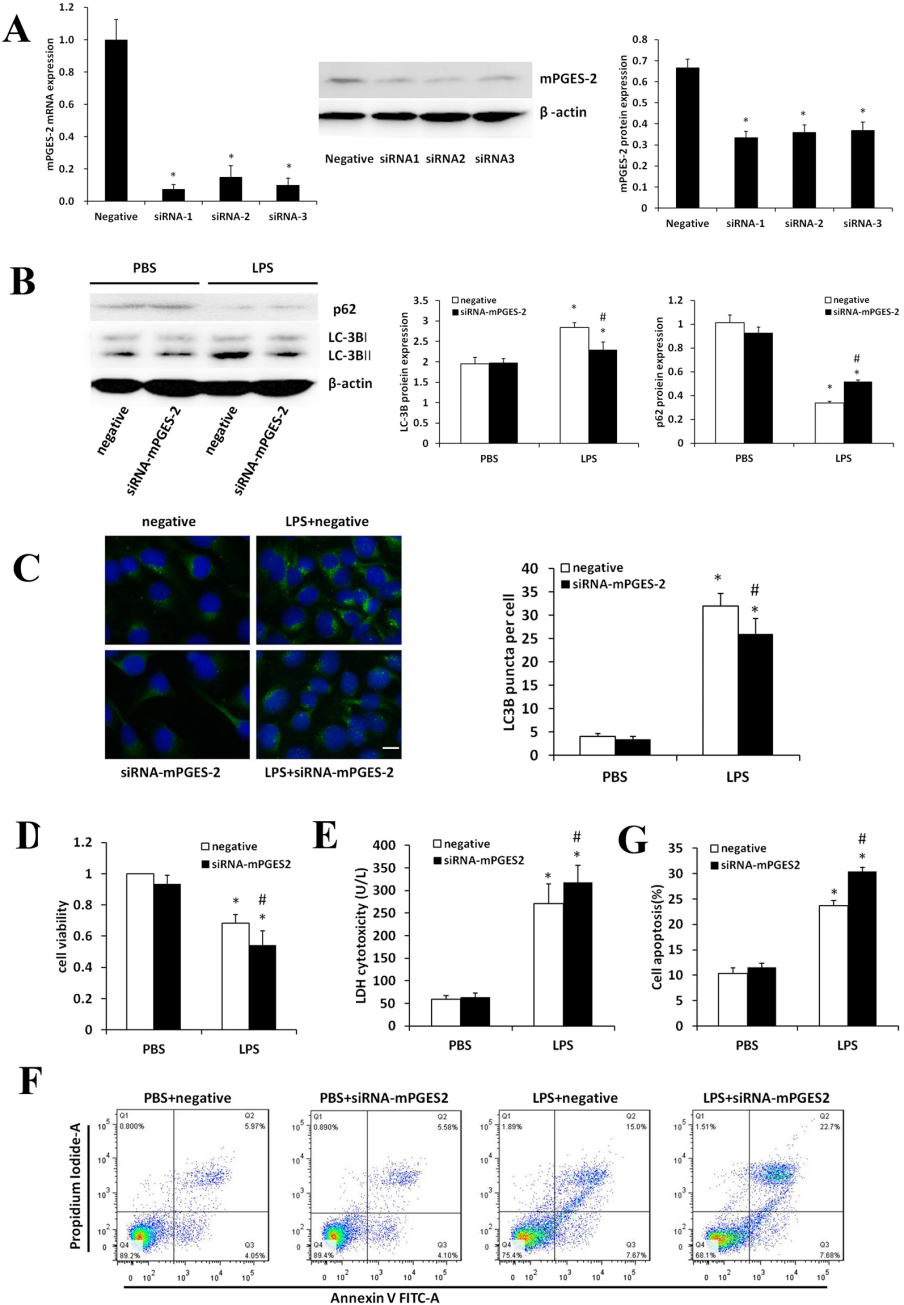
Effects of mPGES-2 interference on autophagy and apoptosis of HK-2 cells treated with LPS. (**A**) Shown are the levels of mPGES-2 expression in HK-2 cells transfected with control siRNA and siRNA-*mPGES-2* after treatment with 1000 ng/ml LPS for 12 h measured by qRT-PCR (left panel) and immunoblot analyses (middle panel). Right panel shows the ratios of mPGES-2/β-actin. (**B**) Shown are the immunoblot results of LC-3B and p62 (left panel) and the ratios of LC3B-II/LC3B-I and p62/β-actin (right panel) in HK-2 cells transfected with control siRNA and siRNA-*mPGES-2* after treatment with 1000 ng/ml LPS for 12 h. (**C**) Shown are the immunofluorescence analyses of HK-2 cells in different groups treated with 1000 ng/mL LPS for 12 h (left panel, scale bar: 20 μm) and the average number of autophagosomes per cell measured in at least 30 cells (right panel). (**D**) Shown are the viabilities of HK-2 cells in different groups measured using CCK8 analysis. (**E**) Shown are the LDH cytotoxicity detection in different groups. (**F**) Shown are the apoptosis of HK-2 cells in different groups examined by flow cytometry. (**G**) Shown are the ratios of apoptotic cells in different groups (right panel). *P < 0.05 versus the control group. ^#^*P* < 0.05 versus the LPS + control plasmid group. Data are mean ± SD. (n = 3).

Additionally, the Supplementary Information file published with this Article contains an error in Supplementary Figure 1. In panels A and D, the label ‘LC-3B’ should read ‘LC-3BI’ and ‘LC-3BII’. In panel F, ‘LPS’ should read ‘H_2_O_2_’. The correct Figure S1 appears below as Figure 2.

**Figure 2:**
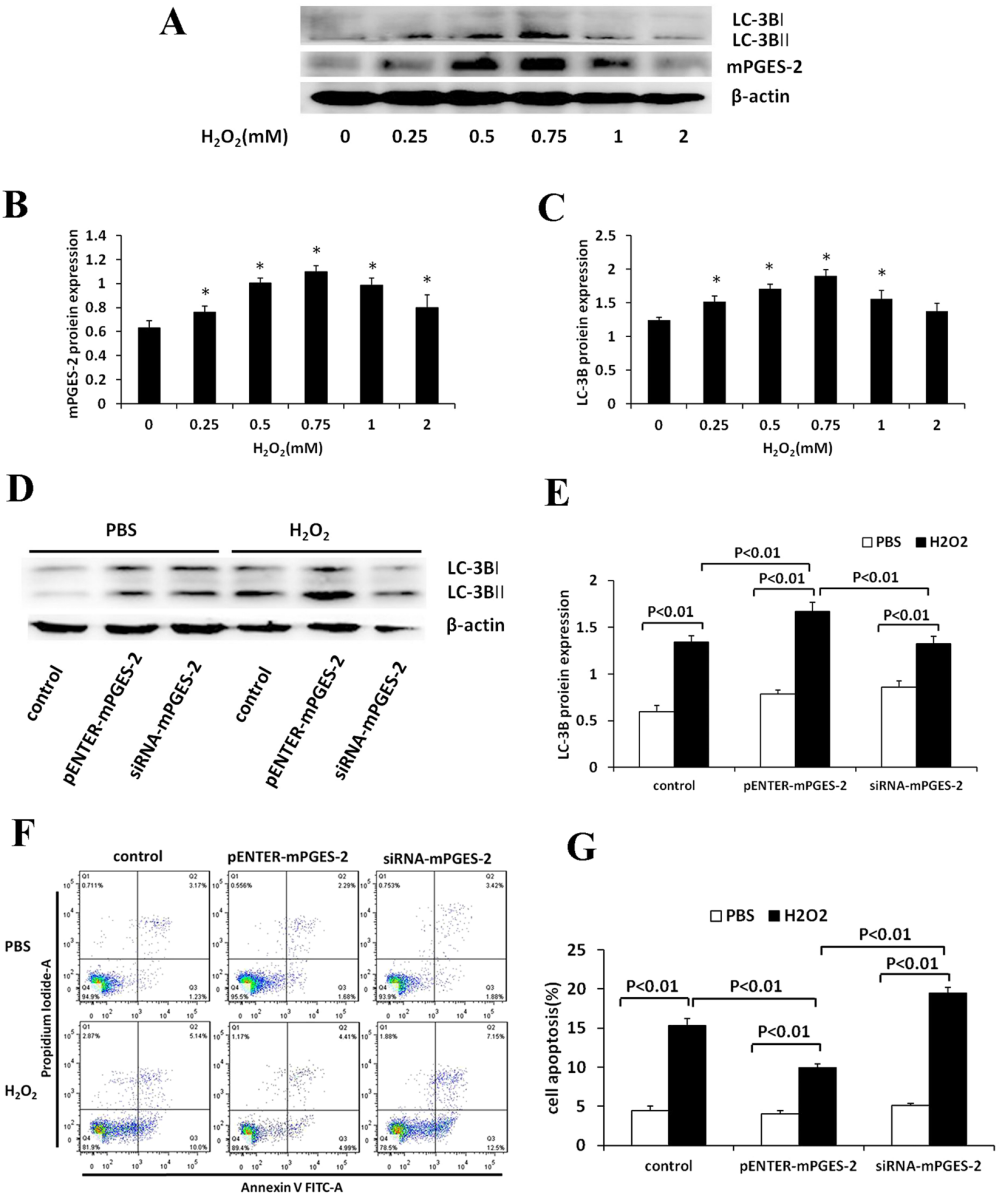
The expression of mPGES-2 in H_2_O_2_-treated HK-2 cells and the effects of mPGES-2 overexpression and interference on autophagy and apoptosis of HK-2 cells treated with H_2_O_2_. (**A**) The expression of mPGES-2 and LC-3B protein in HK-2 cells treated with H_2_O_2_ at different concentrations for 12h. (**B**) The gray scale ratio of mPGES-2 protein to β-actin. (**C**) The gray scale ratio of LC3B-II/LC3B-I. (**D**) Shown are immunoblot of LC3BII/ LC3B-I in HK-2 cells transfected with mPGES-2 plasmid (pENTER-mPGES-2) and siRNA-mPGES-2 after treatment with H_2_O_2_ (0.75 mM) for 12 h. (**E**) Shown are the gray scale ratios of LC3B-II/LC3B-I. (**F**) Shown are the apoptosis of HK-2 cells in different groups examined by flow cytometry. (**G**) Shown are the ratios of apoptotic cells in different groups. *P < 0.05 versus the 0mM group. Data are mean ± SD (n=3).

