# Supplementary material for: Aggravation of acute kidney injury by mPGES-2 down regulation is associated with autophagy inhibition and enhanced apoptosis
Source: Sci Rep. 2017 Aug 31;7:10247. doi: 10.1038/s41598-017-10271-8 (PMC5579259; doi:10.1038/s41598-017-10271-8)
Supplement: Supplementary file 1 — Supplementary information [file 41598_2017_10271_MOESM1_ESM.pdf]

Aggravation of acute kidney injury by mPGES-2 down regulation is associated with autophagy inhibition and enhanced apoptosis

Ting Li[1,2#], Ying Liu[1,#,*], Jie Zhao[3], Shuying Miao[1], Yunfei Xu[1], Ke Liu[1], Meidong Liu[1], Guiliang Wang[4], Xianzhong Xiao[1,*]

[1] Department of Pathophysiology, Xiangya School of Medicine, Central South University, Changsha 410078, China; [2] Department of Physiology, Changzhi Medical College, Changzhi 046000, China; [3] Department of Neurosurgery, Xiangya Hospital, Central South University, Changsha 410078, China; [4] Department of Digestive Internal Medicine, Gannan Medical University Pingxiang Hospital, Pingxiang 337055, China.

[#] These authors contributed equally to this work.

Supplementary Figures

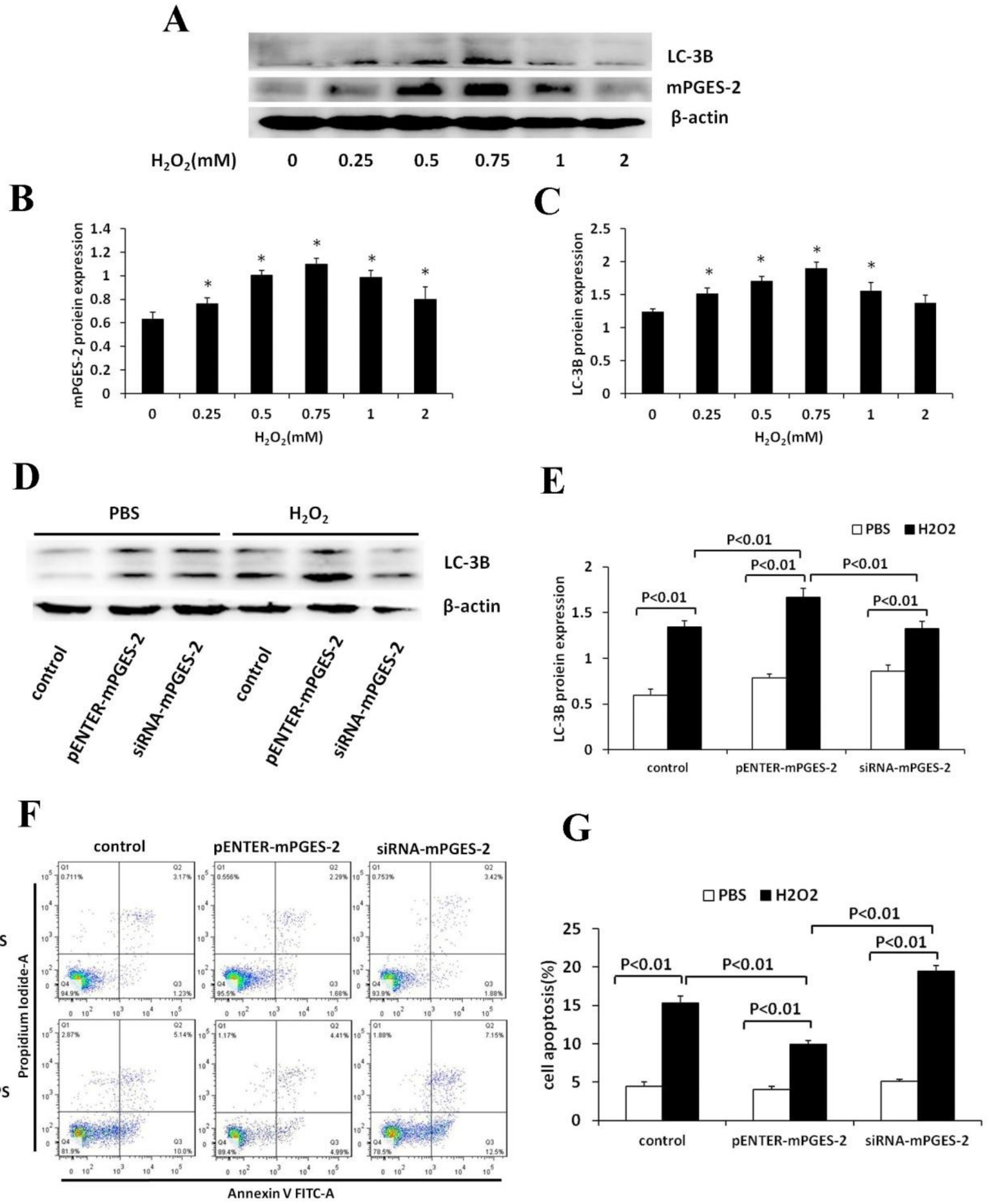

Supplementary Fig.1 The expression of mPGES-2 in H_2O_2-treated HK-2 cells and the effects of mPGES-2 overexpression and interference on autophagy and apoptosis of HK-2 cells treated with H_2O_2

A) The expression of mPGES-2 and LC-3B protein in HK-2 cells treated with H_2O_2 at different concentrations for 12h. B) The gray scale ratio of mPGES-2 protein to

β-actin. C) The gray scale ratio of LC3B-II/LC3B-I. D) Shown are immunoblot of LC3B-II/LC3B-I in HK-2 cells transfected with *mPGES-2* plasmid (pENTER-*mPGES-2*) and siRNA-*mPGES-2* after treatment with H_2O_2 (0.75 mM) for 12 h. E) Shown are the gray scale ratios of LC3B-II/LC3B-I. F) Shown are the apoptosis of HK-2 cells in different groups examined by flow cytometry. G) Shown are the ratios of apoptotic cells in different groups. *P<0.05 versus the 0mM group. Data are mean ± SD (n=3).
